# Phantom earthquake sensations: a cross-sectional analysis of context, perceptual ambiguity, and cognitive intrusion

**DOI:** 10.3389/fpubh.2026.1848434

**Published:** 2026-07-01

**Authors:** Kerem Ersin, Şeyma Tuğba Öztürk

**Affiliations:** 1 Department of Audiology, Faculty of Health Sciences, Istanbul Medipol University, Istanbul, Türkiye; 2 Department of Audiology, College of Heath Sciences, Istanbul Medipol University, Istanbul, Türkiye

**Keywords:** cognitive intrusion, disaster mental health, earthquake anxiety, perceptual ambiguity, phantom earthquake sensations, post-earthquake dizziness, post-traumatic stress, vestibular perception

## Abstract

**Objective:**

Phantom earthquake sensations (PES) are poorly understood. This study investigated relationships between phantom perception, spatial context, earthquake-related anxiety (EAS), and post-traumatic stress (IES-R) following a non-destructive earthquake.

**Methods:**

This cross-sectional study included 157 participants (81 female, 76 male) who experienced the Mw 6.2 Istanbul earthquake. Data were collected within 4 weeks via surveys assessing phantom sensations, EAS, and IES-R. Non-parametric tests, Chi-square, and Spearman rank correlations were used.

**Results:**

Context was a significant modulator. Participants at home reported higher EAS (*p* = 0.037) and IES-R (*p* = 0.033) scores, plus more frequent (*p* = 0.044) and prolonged (*p* = 0.049) phantom sensations. Counter-intuitively, participants perceiving the tremor as “very mild” reported the highest anxiety (Mean = 47), significantly exceeding the “moderate” group (*p* = 0.012), whereas no significant difference was observed with the “severe” group (*p* = 0.609). EAS correlated weakly with total IES-R (*ρ* = 0.283, *p* = 0.033) and moderately with the “intrusion” subscale (ρ = 0.375, *p* = 0.004).

**Conclusion:**

PES is a complex biopsychosocial phenomenon shaped by spatial context (home/solitude) and perceptual ambiguity, rather than by seismic intensity alone. The link to anxiety appears driven by cognitive re-experiencing (intrusion) over generalized fear. Findings support a clinical triad of vestibular rehabilitation, anxiety management, and trigger control.

## Introduction

Beyond their structural devastation, earthquakes inflict profound psychological and physiological consequences, with survivors often reporting persistent anxiety, dizziness, and impaired balance (1, 2). A growing body of research highlights that these aftereffects include a spectrum of illusory experiences, most notably ‘phantom earthquake sensations (PES)’—a persistent feeling of swaying in the absence of measurable seismic activity. In this manuscript, we use PES as an umbrella term that encompasses, but is broader than, the narrower clinical entity ‘Post-Earthquake Dizziness Syndrome (PEDS)’ defined by Miwa and colleagues (3) as brief body-sway illusions lasting less than 1 min within 3 months of the earthquake. Documented in the aftermath of significant events like the 2011 Tōhoku, 2016 Kumamoto, and 2020 Zagreb–Banovina earthquakes (4, 5). These sensations are distinct from aftershock-induced disequilibrium. They can persist for days or weeks and are often accompanied by significant distress, hypervigilance, and behavioral changes (6). This phenomenon suggests that earthquake perception is not merely a response to external ground motion but a complex, multisensory, and context-dependent process modulated by psychological and neurophysiological factors that shape bodily awareness and risk appraisal.

The phantom earthquake phenomenon shows notable parallels with other illusory motion syndromes, such as mal de débarquement syndrome (MdDS), which is characterized by persistent swaying sensations following passive movement exposures, such as sea or air travel (7). In both conditions, the absence of external motion is accompanied by false vestibular feedback and increased autonomic arousal. Case reports have demonstrated that phantom earthquake syndrome can present with vertigo, motor instability, nausea, and vegetative symptoms, frequently misdiagnosed as benign paroxysmal positional vertigo (BPPV) or psychogenic dizziness (6). Importantly, vestibular rehabilitation and multisensory recalibration have been reported to alleviate such symptoms when pharmacological interventions are ineffective (6).

Magnitude alone, however, is not the sole determinant of the impact. Research highlights that earthquake-related fear is also closely associated with individual predisposition, prior trauma, perceptions of building safety, and media exposure. Earthquakes of 4.0 magnitude and above are significant psychological triggers, especially when they are repetitive, occur at night, or are combined with unsafe environmental conditions. This effect can be further strengthened by factors such as an individual’s personal history, the community’s collective trauma, and misinformation. Earthquakes of 4.0 magnitude and higher, especially in cases of repeated tremors, can lead to psychiatric effects such as post-traumatic stress disorder (PTSD), anxiety, and depression. For instance, a study conducted after the 2016 Ecuador earthquake identified depression in 29% of adolescents, anxiety in 15%, and suicidal ideation in 20% (8). This impact is not limited to those directly affected; anxiety levels can increase even in individuals who did not directly experience the earthquake, driven by threat perception and uncertainty. This indicates that even earthquakes in the 4.0–5.0 magnitude range can create fear at a societal level (9).

The vestibular system does not function in isolation; instead, it integrates with visual, somatosensory, and cortical networks to maintain postural stability and spatial orientation. Earthquake-induced low-frequency vibrations (0.1–3.5 Hz) have been shown to disrupt this integration, creating mismatches between expected and actual sensory input (10, 11). Neuroimaging evidence from related phantom phenomena suggests that cortico-limbic-striatal circuits, the entorhinal cortex, the amygdala, and multimodal association areas play central roles in generating or suppressing illusory perceptions (7, 12–14). Thus, phantom earthquake sensations may arise when traumatic memories, heightened vigilance, and disrupted vestibular–visual integration converge, leading to persistent distortions of bodily motion perception.

Despite increasing reports, phantom earthquake sensations remain poorly defined and are often conflated with dizziness or vertigo of peripheral origin (15, 16). This creates diagnostic uncertainty and may delay the implementation of appropriate interventions. There is a critical need to disentangle the psychological, vestibular, and multisensory mechanisms underlying earthquake-related illusory sensations. In this context, our study investigates how phantom earthquake perception emerges under specific conditions (e.g., being at home or alone), its relationship to earthquake anxiety and post-traumatic stress symptoms, and whether it overlaps with classical dizziness complaints. We hypothesize that participants’ responses to questions about their experiences with earthquakes will reveal significant associations between phantom earthquake perception, earthquake anxiety (as measured by the Earthquake Anxiety Scale, EAS), and post-traumatic stress reactions (as measured by the Impact of Event Scale–Revised, IES-R).

On April 23, 2025, a moment magnitude (Mw) 6.2 earthquake occurred off the coast of Silivri in the Sea of Marmara, approximately 40 km west of Istanbul (17). Although the event did not cause widespread structural collapse or mass casualties, it was strongly felt across Istanbul and neighboring provinces, where over 16 million people reside. Preliminary reports from the Turkish Disaster and Emergency Management Authority (AFAD) and the Ministry of Environment, Urbanization, and Climate Change documented limited but non-trivial damage: a small number of unreinforced masonry buildings sustained partial wall collapses, several older structures in Fatih and Küçükçekmece districts were temporarily evacuated, and minor injuries were reported during panic-related evacuations rather than from building failure itself. No fatalities directly attributable to structural collapse were recorded (17).

Despite the limited physical destruction, the psychological impact on the population was substantial. The event reactivated the collective memory of the February 6, 2023, Kahramanmaraş earthquake doublet (Mw 7.8 and 7.5), which had caused more than 50,000 deaths in southeastern Türkiye and remained a vivid reference point in national consciousness (15). Within hours, the government activated rapid damage assessment teams, opened temporary shelter areas, and the Ministry of Health expanded psychosocial support hotlines. Public attention was also amplified by intensive media and social media coverage, including continuous broadcasts of structural inspections, aftershock alerts, and expert commentary on the long-anticipated “Marmara Earthquake.” This context of heightened collective anxiety, governmental visibility, and informational saturation forms the immediate backdrop against which phantom earthquake sensations were experienced and reported in our sample.

## Methods

### Participants

All participants were informed about the aim and scope of the research and provided online informed consent to participate. The study was conducted in accordance with the Declaration of Helsinki and received approval from the Istanbul Medipol University Non-Interventional Clinical Research Ethics Committee (Decision No: 668, June 19, 2025).

A total of 157 voluntary participants (81 females, 76 males; aged 18–65 years) were included in the study. All had personally experienced the April 23, 2025, earthquake originating off Silivri in the Sea of Marmara (Istanbul region), which had a moment magnitude (Mw) of 6.2 and did not result in significant structural damage or injuries.

Inclusion criteria were being at least 18 years of age, having personally felt the earthquake, to provide consistent responses, and having no known psychiatric or neurological disorders. Exclusion criteria included sustaining severe physical injury during the event, experiencing structural collapse of one’s home, or submitting incomplete or inconsistent questionnaire data. Notably, the April 23, 2025 event did not result in major structural collapse in Istanbul. No participants in our sample reported severe structural damage to their residence, and thus no exclusions were made on this basis.

All data were collected either through short face-to-face street interviews or via an online survey form. For the online component, responses were recorded anonymously on a secure platform without personal identifiers (e.g., names, contact information, or IP addresses). For the street interviews, participants were not audio- or video-recorded, and responses were noted anonymously on paper forms. Participation was entirely voluntary, and all participants were informed that they could withdraw at any stage without consequence.

### Sample size

Sample size was determined *a priori* using G*Power 3.1.9.7 (22). Because the principal analyses involved between-group comparisons of non-normally distributed continuous outcomes (EAS and IES-R total scores) across three or more contextual subgroups (e.g., earthquake location: home / workplace / vehicle), the calculation was based on the nonparametric Kruskal–Wallis H test, using its asymptotic relative efficiency of 0.955 versus one-way ANOVA. With a medium effect size (Cohen’s *f* = 0.25, corresponding to ε^2^ ≈ 0.06), *α* = 0.05 (two-tailed), statistical power (1 − *β*) = 0.80, and three independent groups, the minimum total sample size required was 151 (≈ 51 per group). To accommodate up to 5% incomplete or inconsistent responses, recruitment continued until 157 valid cases were obtained. The same parameters comfortably exceed requirements for the secondary correlation analyses (n = 84 required to detect *ρ* = 0.30 at *α* = 0.05, power = 0.80) and the two-group Mann–Whitney U comparisons. As all analyses were exploratory in nature, no formal correction was applied to the omnibus tests for the family-wise error rate; however, Dunn’s test with Bonferroni adjustment was used for pairwise post-hoc comparisons following significant omnibus Kruskal–Wallis tests (see Statistical Analysis section).

### Design and conditions

This study employed a descriptive and cross-sectional design. Data were collected within 4 weeks following the earthquake through a combination of semi-structured street interviews and an online survey. Participants were first asked about their earthquake experiences and behavioral responses, after which two psychometric scales were administered. The study was conducted entirely through observational and questionnaire-based methods, with no experimental manipulation or clinical intervention.

The primary outcomes were defined as two specific measured characteristics of phantom earthquake sensations: (i) the frequency of phantom sway experiences (categorized as: not experienced / occasionally / once a day / several times a day) and (ii) the duration for which participants reported still feeling shaking after the earthquake (categorized as: none / only the first day / 2–3 days / from time to time). The secondary outcomes were defined as the total scores on the Impact of Event Scale–Revised (IES-R) and the Earthquake Anxiety Scale (EAS), together with the three individual IES-R subscale scores (Intrusion, Avoidance, and Hyperarousal). Critically, although phantom duration and frequency served as primary continuous outcomes used to profile the core phenomenon, they were also treated as independent grouping variables in specific cross-tabulated contingency analyses and multi-group secondary outcome comparisons.

Spatial-contextual factors (earthquake location: home / workplace / vehicle), the perceived intensity of the tremor (categorized as: severe / moderate / very mild / did not notice), behavioral responses during the event, the presence of dizziness within the first 24 h, and social media checking behavior were utilized as explanatory or contextual independent variables.

### Questionnaires

Three instruments were used in the study: an earthquake experience and perception questionnaire developed by the researchers based on relevant literature, the Impact of Event Scale–Revised (IES-R) to assess post-traumatic stress reactions, and the Earthquake Anxiety Scale (EAS) to evaluate earthquake-related anxiety.

The earthquake experience and perception questionnaire was constructed to explore the behavioral, perceptual, and contextual aspects of phantom earthquake perception. The items were developed based on prior literature on post-seismic motion illusions and trauma-related perceptual phenomena, aligned with the study hypothesis. The questionnaire examined the context of the earthquake (home, workplace, vehicle), the perceived intensity, behavioral responses during the event (e.g., waiting vs. evacuating), and post-earthquake reactions such as dizziness, a continued sensation of shaking, sleep difficulty, and checking social media. It also assessed the duration and frequency of the phantom sensation, as well as the situational context in which it occurred (e.g., while alone at home, in quiet environments, or while resting). This instrument aimed to characterize the temporal and contextual dimensions of the phantom perception.

The Impact of Event Scale–Revised (IES-R), initially developed by Weiss and Marmar and adapted to Turkish by Çorapçıoğlu et al., consists of 22 items across three subscales: intrusion, avoidance, and hyperarousal. Each item is rated on a 5-point Likert scale ranging from 0 (“not at all”) to 4 (“extremely”). Higher scores indicate greater severity of post-traumatic stress reactions.

The Earthquake Anxiety Scale (EAS) was developed by Bal (21). This 34-item, single-factor instrument was specifically designed to assess earthquake-related anxiety among Turkish individuals. Items are rated on a 5-point Likert scale (1 = “not at all,” 5 = “very much”), with higher total scores reflecting higher earthquake-related anxiety.

Identical item wording and identical instruments were used in both the street-interview and online survey modes; only the administration medium differed (paper form vs. secure web platform). To examine whether the mode of administration biased the principal psychometric outcomes, a post-hoc Mann–Whitney U test compared total EAS and IES-R scores between the two modes. No statistically significant differences were observed between the administration groups (EAS: U = 2894.5, *p* = 0.542; IES-R: U = 3012.0, *p* = 0.816), indicating that the data collection mode did not systematically affect the secondary outcomes.

### Statistical analysis

**Figure 1:**
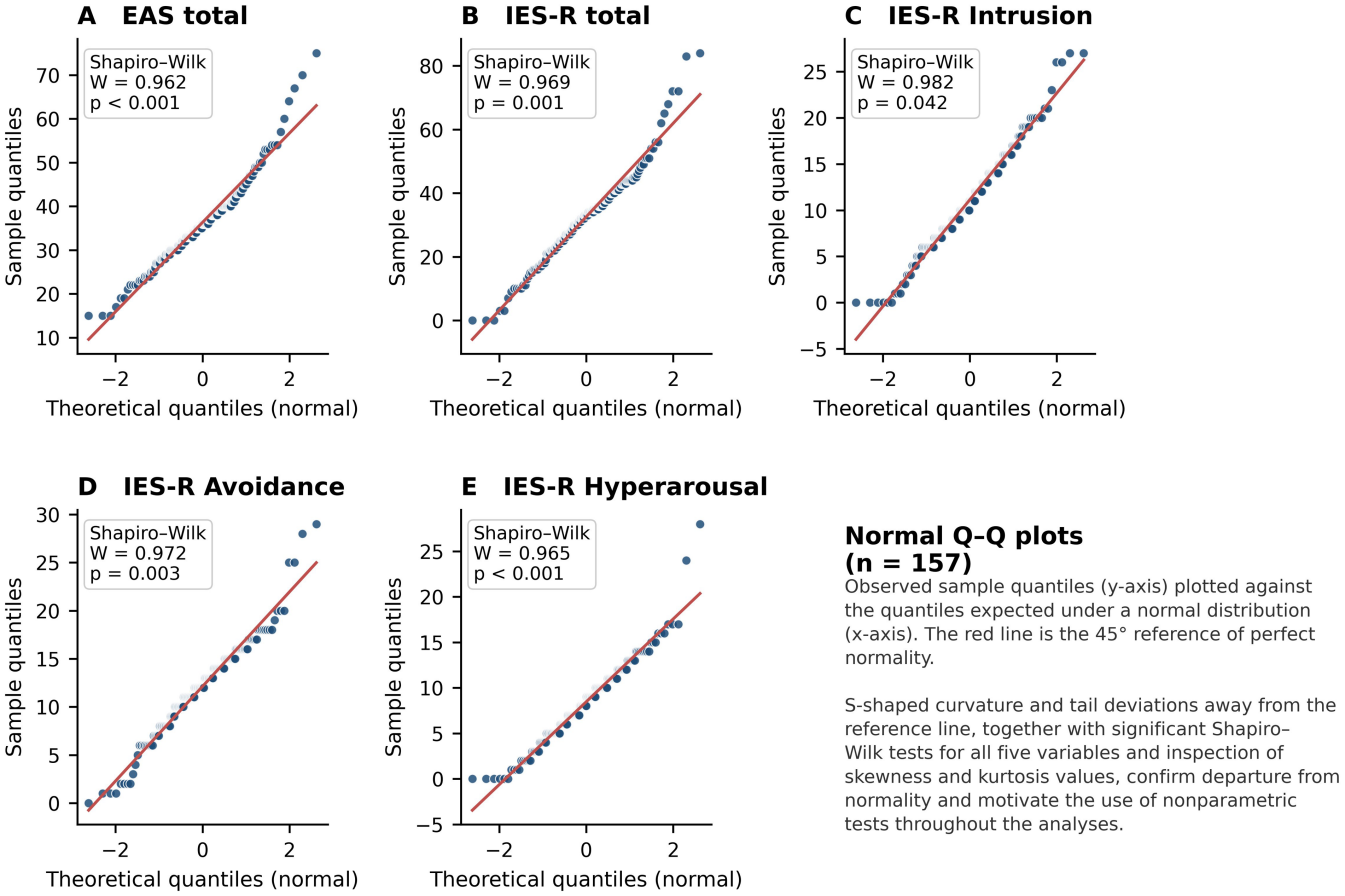
Normal Q–Q plots for EAS total and IES-R total/subscale scores.

All statistical analyses were performed using IBM SPSS Statistics version 27.0. The distributions of the total EAS score, total IES-R score, and the three IES-R subscale scores (Intrusion, Avoidance, and Hyperarousal) were evaluated using multiple complementary approaches: (i) the Shapiro–Wilk test, (ii) visual inspection of histograms and normal Q–Q plots, and (iii) skewness and kurtosis values, where absolute values exceeding |2| were considered indicative of substantive non-normality (West et al.). The Shapiro–Wilk test demonstrated significant deviations from normality for all primary indices, which was further supported by the visual inspection of the normal probability plots and descriptive distribution metrics (Figure 1). Because these criteria indicated a consistent deviation from normality for all primary continuous variables, nonparametric tests were employed in all subsequent group comparisons and bivariate analyses.

Group differences in IES-R and EAS scores based on categorical variables (e.g., earthquake location, perceived intensity, presence of dizziness, and duration of phantom sensations) were examined using the Kruskal–Wallis test for multiple-group comparisons and the Mann–Whitney U test for two-group comparisons. For statistically significant omnibus Kruskal–Wallis outcomes, post-hoc pairwise comparisons were executed using Dunn’s test with automated Bonferroni correction to adjust for multiple comparisons. Associations between categorical variables were assessed using contingency table analyses, incorporating Pearson χ^2^, Likelihood Ratio χ^2^, and Linear-by-Linear Association where appropriate, alongside Cramér’s V to determine effect sizes. Bivariate relationships between continuous psychometric scores were evaluated using Spearman’s rank correlation analysis (*ρ*). Statistical significance was set *a priori* at *p* < 0.05.

Descriptive statistics were presented as mean ± standard deviation (SD). Group differences in IES-R and EAS scores based on independent variables (e.g., earthquake location, perceived intensity, presence of dizziness, duration of phantom sensation) were examined using the Kruskal–Wallis test for multiple-group comparisons and the Mann–Whitney U test for two-group comparisons. When significant differences were identified, Bonferroni-adjusted pairwise post-hoc tests were conducted. Associations between categorical variables were assessed using Crosstabs (Chi-square tests), including Pearson χ^2^, Likelihood Ratio χ^2^, and Linear-by-Linear Association where appropriate. Relationships between questionnaire scores were evaluated using Pearson correlation analysis. Statistical significance was set at *p* < 0.05, and for multiple comparisons, the threshold was adjusted to *p* < 0.017.

## Results

### Descriptive statistics

A total of 157 participants (81 females, 76 males; aged 18–65 years) were included in the analyses. The mean total score on the IES-R was 30.98 ± 12.61, and the mean total score on the EAS was 34.51 ± 8.57. Among the IES-R subscales, mean scores were 10.86 ± 5.74 for *Intrusion*, 11.65 ± 5.00 for *Avoidance*, and 8.47 ± 4.12 for *Hyperarousal*. Overall, participants demonstrated mild-to-moderate levels of post-traumatic stress symptoms and earthquake-related anxiety. Internal consistency reliability in the present sample was assessed via Cronbach’s alpha. The EAS demonstrated excellent reliability (alpha = 0.92, 34 items). The IES-R total scale also showed excellent reliability (alpha = 0.91, 22 items), with individual subscale alphas demonstrating strong internal consistency across all subdimensions: alpha = 0.86 for Intrusion (7 items), alpha = 0.82 for Avoidance (8 items), and alpha = 0.79 for Hyperarousal (7 items). All psychometric instruments met the conventional structural threshold of alpha > 0.70 for research applications.

### Multiple and pairwise comparisons

Group comparisons based on the environment in which participants experienced the earthquake revealed statistically significant differences in both total IES-R (*p* = 0.033) and total EAS scores (*p* = 0.037). The results of these comparisons are summarized in Tables 1, 2. Follow-up post-hoc analyses using Dunn’s test with Bonferroni correction indicated that participants who experienced the earthquake at home reported significantly higher stress (*p* = 0.028) and anxiety scores (*p* = 0.028) than those who were at work. In contrast, no statistically significant pairwise differences were observed among participants who were in a vehicle (*p* > 0.05).

**Table 1:** IES-R total by survey factors.

Factor	Group	Median (IQR)	Intergroup comparison *p*	Pairwise *p*
Earthquake location	Home (a)	34 (17)	0.033*	a-b: 0.028* a-c: 0.321 b-c: 1.000
	Workplace (b)	29 (17)		
	In vehicle (c)	30^a^		
Perceived intensity	Severe (a)	35 (14)	0.463	-
	Moderate (b)	29.5 (22)		
	Very mild (c)	34.5 (14)		
	Did not notice (d)	30^a^		
Dizziness in the first 24 h	Yes (a)	30.5 (11.5)	0.581	-
	No (b)	32 (21.5)		
“Still felt shaking?” duration	No (a)	26 (19.5)	0.222	-
	First day (b)	24 (13.25)		
	2–3 days (c)	34 (13.5)		
	From time to time (d)	36 (11)		

a IQR could not be computed for the “In vehicle” and “Did not notice” subgroups due to insufficient sample size within these specific options.

*p < 0.05; IES-R, Impact of Event Scale–Revised; IQR, Interquartile Range. Pairwise p-values for multi-group comparisons reflect the automated Dunn-Bonferroni adjusted significance values from the SPSS Model Viewer.

**Table 2:** EAS (Earthquake Anxiety Scale) total by survey factors.

Factor	Group	Median (IQR)	General *p*-value	Pairwise Comparisons
Earthquake location	Home (a)	35 (10)	0.037*	a-b: 0.028* a-c: 0.258 b-c: 1.000
	Workplace (b)	29 (10)		
	In vehicle (c)	24^a^		
Perceived intensity	Severe (a)	36 (9)	0.011*	a-b: 0.370 a-c: 0.609 a-d: 1.000 b-c: 0.012* b-d: 1.000 c-d: 0.699
	Moderate (b)	31 (10.25)		
	Very mild (c)	47 (22)		
	Did not notice (d)	32^a^		
Dizziness in the first 24 h	Yes (a)	41.5 (14.5)	0.621	-
	No (b)	33 (9.5)		
“Still felt shaking?” duration	No (a)	35 (13.5)	0.128	-
	First day (b)	29 (9.75)		
	2–3 days (c)	36 (10)		
	From time to time (d)	33 (13)		

a IQR could not be computed for the “In vehicle” and “Did not notice” subgroups due to insufficient sample size within these specific options.

*p < 0.05; EAS, Earthquake Anxiety Scale; IQR, Interquartile Range. Pairwise *p*-values for multi-group comparisons reflect the automated Dunn-Bonferroni adjusted significance values from the SPSS Model Viewer.

Comparisons according to the perceived intensity of the earthquake revealed no statistically significant differences in total IES-R scores (*p* = 0.463), whereas statistically significant differences were observed in total EAS scores (*p* = 0.011). Post-hoc pairwise analyses showed that participants who perceived the earthquake as “Very mild” reported significantly higher anxiety scores (Median = 47) than those who perceived it as “Moderate” (Median = 31, adjusted *p* = 0.012). However, following Bonferroni correction, the difference in anxiety scores between the “Very mild” and “Severe” groups, as well as all remaining pairwise comparisons, was not statistically significant (*p* > 0.05).

When participants were grouped according to whether they experienced dizziness within the first 24 h after the earthquake, no statistically significant differences were observed in either total IES-R scores (*p* = 0.581; Table 1) or total EAS scores (*p* = 0.621; Table 2).

Finally, analyses based on the duration of the perceived shaking sensation revealed no statistically significant differences in either total IES-R scores (*p* = 0.222) or total EAS scores (*p* = 0.128). Therefore, no follow-up pairwise comparisons were conducted for these variables.

### Correlation analyses

The relationships between the IES-R subscales (Intrusion, Avoidance, and Hyperarousal) and the total scores of both the IES-R and the EAS were examined using Spearman rank correlation analysis. The results are presented in Table 3.

**Table 3:** Spearman rank correlations between IES-R subscales and EAS total score.

Variable	Intrusion	Avoidance	Hyperarousal	IES-R total	EAS total
Median (IQR)	10 (8.5)	13 (7)	9 (6.5)	32 (21)	34 (12)
IES-R Intrusion	—	ρ = 0.543 *p* < 0.001**	ρ = 0.623 *p* < 0.001**	ρ = 0.874 *p* < 0.001**	ρ = 0.375 *p* = 0.004**
IES-R Avoidance	ρ = 0.543 *p* < 0.001**	—	ρ = 0.572 *p* < 0.001**	ρ = 0.830 *p* < 0.001**	ρ = 0.186 *p* = 0.165
IES-R Hyperarousal	ρ = 0.623 *p* < 0.001**	ρ = 0.572 *p* < 0.001**	—	ρ = 0.837 *p* < 0.001**	ρ = 0.116 *p* = 0.389
IES-R total	ρ = 0.874 *p* < 0.001**	ρ = 0.830 *p* < 0.001**	ρ = 0.837 *p* < 0.001**	—	ρ = 0.283 *p* = 0.033*
EAS total	ρ = 0.375 *p* = 0.004**	ρ = 0.186 *p* = 0.165	ρ = 0.116 *p* = 0.389	ρ = 0.283 *p* = 0.033*	—

*p ≤ 0.05; **p ≤ 0.01; IES-R, Impact of Event Scale–Revised; EAS, Earthquake Anxiety Scale; IQR, Interquartile Range; ρ, Spearman correlation coefficient.

All IES-R subscales demonstrated moderate-to-strong positive correlations with one another and strong positive correlations with the total IES-R score (*ρ* = 0.543–0.874, *p* < 0.001), indicating a high degree of internal consistency among the post-traumatic stress dimensions. In addition, the total EAS score showed a weak but statistically significant positive correlation with the total IES-R score (*ρ* = 0.283, *p* = 0.033).

Among the IES-R subscales, only the Intrusion subscale was significantly associated with earthquake-related anxiety (ρ = 0.375, *p* = 0.004). No statistically significant correlations were observed between the total EAS score and the Avoidance (ρ = 0.186, *p* = 0.165) or Hyperarousal (ρ = 0.116, *p* = 0.389) subscales.

### Associations between contextual variables and phantom perception indicators

The relationship between the environment in which participants experienced the earthquake and the duration of the subsequent phantom shaking sensation was examined. Associations between earthquake-related variables and phantom perception indicators were evaluated using contingency analyses, and the results are presented in Table 4.

**Table 4:** Crosstab analyses between earthquake-related variables and phantom perception indicators.

Contextual explanatory factor	Phantom outcome variable categories	n	Row %	Statistical metric	df	*p*-value	Cramer’s V
Earthquake location	“Still felt shaking” duration			Likelihood Ratio χ^2^ = 12.632	6	0.049*	0.201
*Home*	No	7	17.9%				
	First day	7	17.9%				
	2–3 days	16	41.0%				
	Time to time	9	23.1%				
*Workplace*	No	6	40.0%				
	First day	4	26.7%				
	2–3 days	1	6.7%				
	Time to time	4	26.7%				
*In vehicle*	No	0	0.0%				
	First day	1	33.3%				
	2–3 days	0	0.0%				
	Time to time	2	66.7%				
Earthquake location	Frequency of feeling the motion			Likelihood Ratio χ^2^ = 15.906	8	0.044*	0.225
*Home*	Rarely	8	20.5%				
	Once a day	6	15.4%				
	Several times a day	19	48.7%				
	Several times a week	4	10.3%				
	Still feeling	2	5.1%				
*Workplace*	Rarely	9	60.0%				
	Once a day	0	0.0%				
	Several times a day	2	13.3%				
	Several times a week	3	20.0%				
	Still feeling	1	6.7%				
*In vehicle*	Rarely	1	33.3%				
	Once a day	0	0.0%				
	Several times a day	2	66.7%				
	Several times a week	0	0.0%				
	Still feeling	0	0.0%				
Perceived intensity	Context where sensation occurred			Linear-by-Linear χ^2^ = 3.980	1	0.046*	0.159
*Severe*	While lying down	3	20.0%				
	Crowded environment	2	13.3%				
	Quiet environment	2	13.3%				
	Alone at home	8	53.3%				
*Moderate*	While lying down	2	5.9%				
	Crowded environment	5	14.7%				
	Quiet environment	5	14.7%				
	Alone at home	22	64.7%				
*Very mild*	While lying down	0	0.0%				
	Crowded environment	0	0.0%				
	Quiet environment	1	16.7%				
	Alone at home	5	83.3%				
*Did not notice*	While lying down	0	0.0%				
	Crowded environment	0	0.0%				
	Quiet environment	0	0.0%				
	Alone at home	2	100.0%				
Behavioral response	Social media check in first 24 h			Pearson χ^2^ = 4.643	1	0.031*	0.172
*I went outside*	Yes	6	15.0%	*(Fisher’s Exact p = 0.043)*			
	No	34	85.0%				
*I waited*	Yes	7	41.2%				
	No	10	58.8%				

n represents the absolute frequency within rows; Row % indicates the percentage calculated within each specific category of the explanatory factor. The total valid sample analyzed for these subsets was *n* = 57. *p < 0.05 indicates statistical significance. Due to the high proportion of cells with expected counts less than 5, the Likelihood Ratio, Linear-by-Linear Association, and Fisher’s Exact tests were systematically preferred and reported.

The Likelihood Ratio test revealed a statistically significant association between earthquake location and phantom shaking duration (χ^2^(6) = 12.633, *p* = 0.049), with a moderate effect size (Cramer’s V = 0.201). This result was interpreted in preference to the Pearson χ^2^ test (*p* = 0.092) due to low expected cell counts (<5) in 66.7% of the contingency table cells. Participants who experienced the earthquake at home were more likely to report phantom shaking lasting 2–3 days than those in other environments.

Similarly, a statistically significant association was observed between earthquake location and the frequency of phantom shaking sensations (Likelihood Ratio χ^2^(8) = 15.910, *p* = 0.044; Pearson χ^2^ *p* = 0.097), with a moderate effect size (Cramer’s V = 0.225). Participants who experienced the earthquake at home were more likely to report experiencing the sensation several times per day, whereas those at work tended to report it only rarely.

The relationship between perceived earthquake intensity and the context in which the phantom sensation occurred was also examined. Although neither the Pearson χ^2^ test nor the Likelihood Ratio test reached statistical significance (*p* = 0.772 and *p* = 0.634, respectively), a statistically significant linear trend was identified (Linear-by-Linear Association, χ^2^(1) = 3.982, *p* = 0.046), indicating a small-to-moderate effect size (Cramer’s V = 0.159). Participants who perceived the earthquake as more severe were more likely to report experiencing the phantom sensation while alone at home.

Finally, the relationship between behavioral response during the earthquake and social media checking within the first 24 h was examined. A statistically significant association was observed (Pearson χ^2^(1) = 4.641, *p* = 0.031; Fisher’s Exact *p* = 0.043), with a small-to-moderate effect size (Cramer’s V = 0.172). Approximately 41% of participants who reported “I waited” checked social media, compared with 85% of those who reported “I went outside.”

## Discussion

This study shows that the sensation of “feeling as if swaying” after non-destructive earthquakes (the phantom earthquake/PES continuum) is not just a subjective complaint. Instead, it appears to be a phenomenon shaped by spatial context (being at home or alone), perceived intensity, cognitive–autonomic load, and a vestibular–sensory component. In our findings, the “home” context significantly increased both IES-R total and EAS scores (post-hoc: home > workplace), and the most substantial difference in the EAS depended on “how the earthquake was felt,” suggesting that perceptual intensity is sensitive both to context and to interoceptive focus. This pattern aligns with retrospective data reported after major earthquakes, showing impairment in balance function, cVEMP, and HUT abnormalities, which are explained by autonomic stress and sensory interruptions (3).

However, a notable finding regarding perceived intensity warrants discussion. Pairwise comparisons revealed that participants who perceived the earthquake as “Very mild” reported significantly higher anxiety scores than those who perceived it as “Moderate” (*p* = 0.012). In contrast, no statistically significant difference was observed between the “Very mild” and “Severe” groups (*p* = 0.609). This pattern suggests that the primary driver of anxiety may not be the objective physical magnitude of the event, but rather the perceptual ambiguity associated with milder, more difficult-to-interpret sensations. The uncertainty of whether a ‘very mild’ sensation was an earthquake, a structural issue, or an aftershock may create cognitive threat ambiguity that generates more anxiety than a clearly defined, severe event. This ambiguity could lead to a constant re-evaluation of one’s environment and bodily sensations (hypervigilance), thereby increasing anxiety.

While PEDS as defined by Miwa and colleagues (3, 4) refers specifically to brief body-sway illusions lasting <1 min within 3 months after the earthquake, in the present study we adopt the broader term “Phantom Earthquake Sensations (PES)” to encompass the full spectrum of illusory sway experiences—including longer-duration sensations and those modulated by spatial–contextual cues—reported by survivors in our community sample. The literature on PEDS, originally documented after the 2011 Tōhoku and 2016 Kumamoto earthquakes, characterizes the phenomenon as a “body-sway illusion lasting <1 min within 3 months after the earthquake” (3). The proposed mechanism involves low-frequency (0.1–3.5 Hz) vibrations that create conflicts with visual/somatosensory cues and combine with autonomic dysfunction (18). In the Kumamoto earthquake data, factors that increased PEDS prevalence included age (≤21), female sex, high-floor residence, tinnitus/aural fullness, anxiety, autonomic symptoms, and a history of motion sickness. Moreover, the rate of PEDS did not track one-to-one with the number of aftershocks, indicating that higher-level sensory mismatch and expectancy effects come into play (4).

In our data, the weak-to-moderate correlation between the EAS and IES-R total, alongside a stronger correlation only between the EAS and the “re-experiencing” subscale, supports a trajectory driven less by “fear” and more by a “re-experiencing/metacognitive expectancy through vestibular interpretation” pathway. Prior post-earthquake research has shown that aftershock exposure can impair balance under no-visual-compensation conditions and that this impairment is related to state anxiety, suggesting that anxiety and sensory conflict can affect vestibular networks at higher centers (18).

The clinical spectrum of the phantom earthquake phenomenon spans from short PES episodes lasting seconds to minutes to false sway episodes occurring indoors without recorded tremors. A pilot after the Zagreb–Banovina event proposed an operational model; false motion sensations were reported along with vegetative/motor symptoms, marked psychological distress, and functional behavior changes (hypervigilance, avoidance) (5). A case reported from Türkiye described chronic dizziness unresponsive to betahistine that improved markedly within 1 month with vestibular rehabilitation, lifestyle adjustments, and vitamin D, underscoring the primacy of non-pharmacological sensory recalibration (6). The case report also discussed overlaps with MdDS and a possible vitamin D-otolithic contribution (6, 7); this biological axis aligns with our finding that interoceptive focus in the “home/being alone” context amplifies symptoms. Interestingly, experiencing objective dizziness within the first 24 h following the earthquake did not result in a statistically significant difference in either traumatic distress (*p* = 0.581) or anxiety scores (*p* = 0.621) in our cohort. Accordingly, acute transient dizziness immediately after a seismic event did not appear to be a distinguishing factor for long-term psychological distress.

The reason why the place effect (home) is dominant is thought to be that in a quiet/static indoor environment, weak proprioceptive/cue signals are more likely to be distorted by top-down expectations. The perception of seismic accelerations in the vestibular organs—particularly loads on the otoliths in the 0.1–3.5 Hz band—lays the groundwork for misbinding small internal signals as “I am still swaying” (15, 16). Moreover, clusters of balance disturbances cannot be explained solely by “mechanical aftershocks”; sensory conflict, changes in living conditions, and autonomic stress make meaningful contributions to the persistence of phantom sensations in the post-event period (11, 14).

Our findings showed that participants who took an active response during the earthquake, such as ‘I went outside,’ checked social media more frequently in the first 24 h than those who ‘waited.’ This suggests that the group attempting to manage the event actively was also more exposed to threat cues (e.g., damage reports, aftershock warnings) and external validation. This increased exposure may, in turn, enhance hypervigilance and interoceptive focus, thereby contributing to the frequency of phantom sensation episodes. This interpretation is consistent with the role of cognitive/media reinforcement also seen in data from the Kumamoto earthquake. The lack of a one-to-one overlap between PES prevalence and the number of aftershocks in Kumamoto also points to the role of cognitive/media reinforcement (3).

The EAS used shows very high internal consistency, with strong split-half/Spearman–Brown/Guttman reliability; it is a suitable tool for post-earthquake risk screening and intervention monitoring. In addition, the scale has been reported to have a 34-item single-factor structure, with a Likert scoring of 1–5, where higher scores correspond to higher anxiety, a concept that is clearly defined (19).

When read together, the findings and the literature indicate that the “earthquake–vestibular” relationship constitutes a biopsychosocial continuum: (i) peripheral: otolithic/vestibular involvement and low-frequency accelerations (10); (ii) autonomic: HUT positivity and vegetative load; (iii) central/cognitive: the anxiety–re-experiencing–expectancy cycle (12). Although our findings are consistent with the proposed vestibular–autonomic–cognitive triad, the relative weight of peripheral otolithic dysfunction versus central cognitive–emotional processing cannot be adjudicated from self-report data alone, and objective vestibular phenotyping in future cohorts is warranted. Therefore, a hybrid primary-care approach is needed: a detailed earthquake history and triggers; stabilometry and VEMP evaluation in suitable cases; vestibular rehabilitation (gaze stabilization, postural balance, habituation), media/trigger management, and cognitive restructuring; where necessary, selective pharmacotherapy as well as correction of modifiable factors such as vitamin D (6). There is both population-level and case-based evidence that this model may be effective.

PES phenomena lie on a continuum where vestibular mechanisms interact with autonomic stress and cognitive re-experiencing. Our findings show that spatial context (home/being alone), perceptual intensity, and anxiety levels are decisive on this continuum; clinically, the triad of vestibular rehabilitation, anxiety management, and trigger control is a rational target.

### Limitations

Several limitations should be considered when interpreting these findings. First, the cross-sectional design inherently limits our ability to draw causal inferences. Second, the strong ‘home/being alone’ effect we observed may be confounded by unmeasured environmental factors, such as the specific measurement setting, visual background, or momentary noise. Third and most importantly, the present study did not include objective peripheral vestibular function testing (e.g., cervical and ocular vestibular evoked myogenic potentials [cVEMP/oVEMP], video head impulse test [vHIT], caloric irrigation testing, or static and dynamic posturography). All vestibular and dizziness-related variables were assessed exclusively through self-report. Consequently, we cannot disentangle the relative contributions of (i) peripheral otolithic or semicircular canal involvement, (ii) central vestibular–autonomic interactions, and (iii) higher-order cognitive and interoceptive processes to the reported phantom sensations. Although prior post-earthquake studies have documented objective cVEMP, head-up tilt, and posturographic abnormalities (15, 16, 20), our community-based recruitment strategy was deliberately designed to maximize participation in the early post-event window (within 4 weeks) and to minimize self-selection bias that would have arisen from requiring participants to attend an audio-vestibular laboratory.

To address these issues and build upon our findings, future research should move in several key directions. There is a clear need for (i) objective confirmation of daily swaying sensations using in-home passive sensors (accelerometers) and (ii) prospective follow-up studies that combine these real-world data with laboratory-based assessments. Methodologically, the field requires (iii) the establishment of standard operational definitions to distinguish between PEDS (the strict <1-min body-sway illusion) and the broader PES spectrum. Clinically, (iv) randomized controlled trials (RCTs) are necessary to test bundled interventions, such as vestibular rehabilitation combined with media hygiene and cognitive components. Finally, (v) biomarker-supported protocols, particularly those targeting the vitamin D axis and its potential effect on otolith function, represent a promising avenue for mechanistic investigation. Future hybrid studies combining brief community screening with selective vestibular laboratory phenotyping in a high-symptom subgroup represent a critical next step.

Fourth, the distribution of participants across certain categories was heavily skewed. Specifically, a very limited number of individuals experienced the earthquake inside a vehicle or reported that they did not notice the tremor at all. Consequently, stable descriptive metrics such as the Interquartile Range (IQR) could not be computed for the ‘In vehicle’ and ‘Did not notice’ subgroups. This uneven group allocation limits the statistical power of comparisons involving these specific contexts. Future research should employ stratified sampling strategies to ensure a more balanced distribution across all environmental and perceptual categories.

The present analysis focused exclusively on evaluating direct group differences, contingency distributions, and bivariate correlations among the variables. Consequently, potential indirect pathways, confounding networks, or complex interaction mechanisms remain unexamined. For instance, while our findings confirm that earthquake-related anxiety and the intrusion subscale are closely intertwined with phantom sensations, whether anxiety formally mediates the structural trajectory between the initial phantom perception and subsequent long-term post-traumatic stress symptoms could not be modeled. Given the cross-sectional design of this study, any implied directional paths must be interpreted with extreme caution and cannot be utilized as definitive causal evidence. Future longitudinal and prospective research incorporating advanced structural equation modeling (SEM) or longitudinal mediation analysis is warranted to fully disentangle these complex, indirect psychophysiological pathways.

## Conclusion

Phantom Earthquake Sensations (PES) following non-destructive earthquakes constitute a biopsychosocial continuum in which vestibular mechanisms interact with autonomic stress and cognitive re-experiencing. Our findings show that spatial context (being at home / alone), perceptual ambiguity, and the intrusion dimension of post-traumatic stress are key modulators on this continuum. The pattern of associations supports a clinical triad of vestibular rehabilitation, anxiety management, and trigger control as a rational intervention target. Confirmation through hybrid designs combining community-based psychometric screening with objective vestibular phenotyping is the necessary next step.

## Data Availability

The original contributions presented in the study are included in the article/supplementary material, further inquiries can be directed to the corresponding author.
